# Palaeoclimatic conditions in the Mediterranean explain genetic diversity of *Posidonia oceanica* seagrass meadows

**DOI:** 10.1038/s41598-017-03006-2

**Published:** 2017-06-02

**Authors:** Rosa M. Chefaoui, Carlos M. Duarte, Ester A. Serrão

**Affiliations:** 1 CCMAR - Centro de Ciências do Mar, CIMAR Laboratório Associado, Universidade do Algarve, Campus de Gambelas, 8005-139 Faro, Portugal; 2 King Abdullah University of Science and Technology (KAUST), Red Sea Research Center (RSRC), Thuwal, 23955-6900 Saudi Arabia

**Keywords:** Palaeoecology, Phylogenetics, Palaeoclimate

## Abstract

Past environmental conditions in the Mediterranean Sea have been proposed as main drivers of the current patterns of distribution of genetic structure of the seagrass *Posidonia oceanica*, the foundation species of one of the most important ecosystems in the Mediterranean Sea. Yet, the location of cold climate refugia (persistence regions) for this species during the Last Glacial Maximum (LGM) is not clear, precluding the understanding of its biogeographical history. We used Ecological Niche Modelling together with existing phylogeographic data to locate Pleistocene refugia in the Mediterranean Sea and to develop a hypothetical past biogeographical distribution able to explain the genetic diversity presently found in *P. oceanica* meadows. To do that, we used an ensemble approach of six predictive algorithms and two Ocean General Circulation Models. The minimum SST in winter and the maximum SST in summer allowed us to hindcast the species range during the LGM. We found separate glacial refugia in each Mediterranean basin and in the Central region. Altogether, the results suggest that the Central region of the Mediterranean Sea was the most relevant cold climate refugium, supporting the hypothesis that long-term persistence there allowed the region to develop and retain its presently high proportion of the global genetic diversity of *P. oceanica*.

## Introduction

The Mediterranean Sea is a semi-enclosed basin considered a hotspot of biodiversity^1^, harbouring a rich biota which includes a high number of endemic species^2^. Past changes in oceanographic conditions in the Mediterranean Sea have largely influenced the current patterns of biodiversity and genetic structure of its species due to changes in sea level, circulation, and climate forcing across time^3^. In addition, the genetic structure of Mediterranean species has also been shaped by persistent differences between the Western and Eastern basins of the Mediterranean Sea, with the later being more oligotrophic and warmer but less biodiverse than the western basin^1,4^. A genetic boundary between the Eastern and Western Mediterranean Sea has been described for various species including seagrasses^5^, fish^6^, sea cucumbers^7^, crabs^8^ and bivalves (e.g. ref. 9).

*Posidonia oceanica* (L.) Delile is one of the most important Mediterranean endemics, as its meadows form valuable ecosystems providing food, substrate and shelter for other species^10^. The beds of *P. oceanica* are threatened ecosystems, which have been declining mainly in the north-west Mediterranean Sea because of its sensitivity to human impacts, biological invasions and climate change^11–13^, resulting in a loss rate of *P*. *oceanica* density or biomass at −6.9% yr^−1^ in the Mediterranean^14^. A west-east separation has been found in the genetic structure of *P. oceanica* populations, leading to hypothesize an on-going vicariance process^15–17^. These studies found higher allelic richness in the central *P*. *oceanica* populations, around the Siculo-Tunisian Strait, and hypothesized that it might have acted as a genetic boundary during low sea level periods and as a secondary contact zone after the Last Glacial Maximum (LGM ~26000–19000 ya). Given that *P. oceanica* meadows grow very slowly an can persist over millenary time scales^18^, with genotypes possibly exceeding 10,000 years in maximum age^19^, Pleistocene glacial episodes are particularly likely to have affected the current genetic structure and distribution of *P. oceanica*. However, the location of refugia allowing persistence during the most recent cold maximum (the last Pleistocene ice age) remains unclear, leaving uncertainties in the biogeographical history of this foundational seagrass species.

In this study we address these biogeographic questions for *Posidonia oceanica* by using ecological niche modelling (ENM) to infer the hypothetical effects of past climatic conditions on the distribution of genetic diversity of *P. oceanica*, and by comparing the model results with available information on genetic structure of the populations across the species distributional range. ENM combines the known distributional data for the species with predictors (usually environmental and climatic variables) to produce a statistical model transferable to the geographical space. Species distribution models together with phylogeographic data lead to inferences of biogeographical processes (e.g. ref. 20), and have been used to identify climatic refugial areas and postglacial colonization routes^21^. These approaches (ENM coupled with genetic data) applied in the marine realm have revealed the key roles played by glacial refugia in harboring unique high diversity that was left behind as climate-driven range expansions proceeded^22–25^.

In this study, we hindcast the distribution of *P. oceanica* to the conditions during the Last Glacial Maximum (LGM) to identify the putative past glacial refugia and assess their correspondence with the currently known spatial distribution of genetic diversity of the species throughout its range. We hypothesize that glacial refugia areas still harbour the highest genetic diversity across the species range, due to long-term persistence of stable populations. We further hypothesize that regions of reduced genetic diversity found in the extant range of *P. oceanica* correspond to areas affected by range contractions during colder periods in the Mediterranean Sea, as local extinctions and bottlenecks reduce genetic diversity. To test these hypotheses, we produce an ensemble of species distribution models for current conditions and its corresponding projections to the LGM under two different ocean general circulation models (CNRM-CM5 and CCSM4). Our results are then compared with available data on allelic richness and private alleles for *P. oceanica* across the Mediterranean Sea.

## Results

### Ecological niche models of *P. oceanica*

Two uncorrelated variables were retained for the rest of analyses: the minimum SST in winter and the maximum SST in summer. Validation scores obtained for the ensemble for present conditions were: AUC = 0.866; TSS = 0.570; sensitivity = 91.504; specificity = 65.424; while mean validation results for all the models computed were: AUC = 0.813; TSS = 0.526; sensitivity = 87.303; specificity = 66.187 (see Supplementary Table S1 for a description by algorithm).

**Figure 1:**
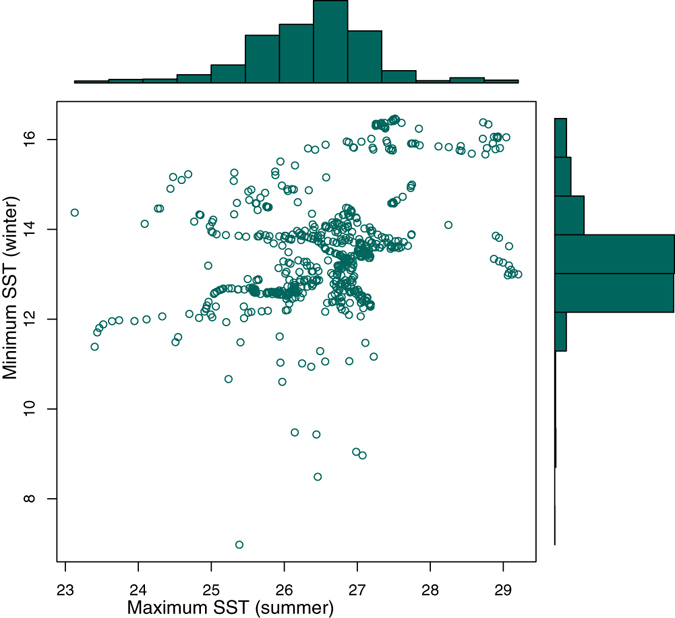
Sea surface temperatures (SST) at which *Posidonia oceanica* meadows are found in the Mediterranean Sea according to the locations compiled in this study. The figure illustrates the high seasonality between summer and winter. Fewer presence records occur in marginal conditions of SST below 10 °C or above 28 °C.

We found that *P. oceanica* occurs from a range of 6.98 °C to 16.47 °C in relation to the minimum winter SST, and from 23.13 °C to 29.21 °C for the maximum summer SST (Fig. 1). The minimum SST in winter obtained a higher relevance score in the ensemble (mean = 0.693) than the maximum SST in summer (mean = 0.486).

**Figure 2:**
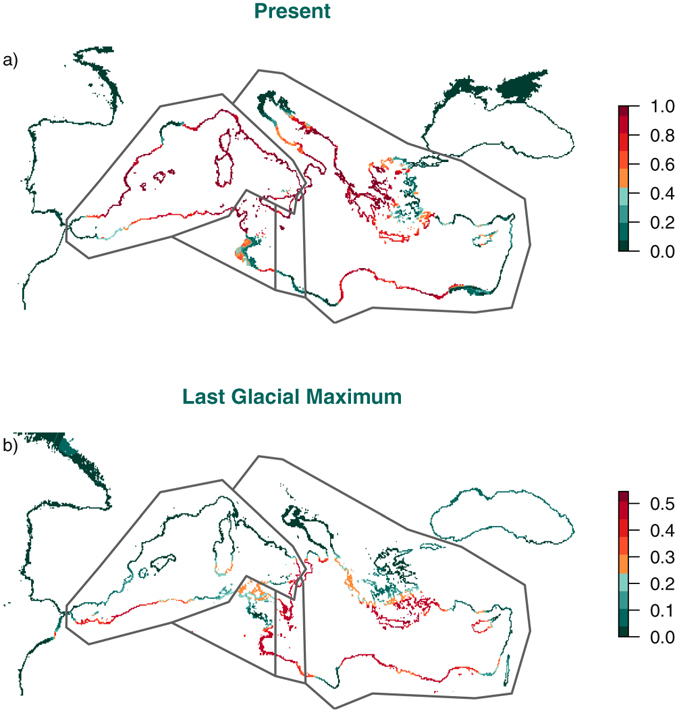
Distribution of *Posidonia oceanica* predicted by ensembles under current (**a**) and Last Glacial Maximum (LGM) conditions (**b**). A major probability of occurrence is predicted in the Western and Northern Mediterranean Sea for the present. Three putative glacial refugia, located in the Southern Mediterranean, were found by hindcasting to the LGM. Divisions correspond to genetic regions identified in the Mediterranean Sea by ref. 15 and ref. 17. Maps were generated using R^43^.

**Figure 3:**
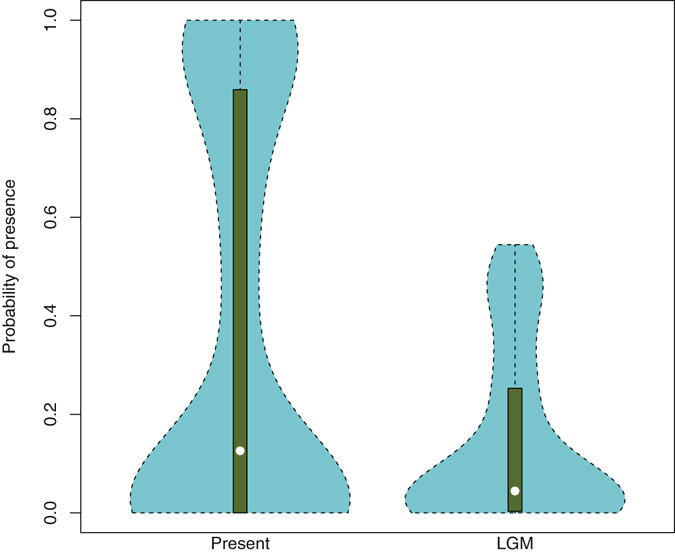
Distribution of probability of presence predicted for *Posidonia oceanica* under current and Last Glacial Maximum (LGM) conditions. Globally, climatic conditions in the Mediterranean Sea were less suitable for *P. oceanica* during the LGM than in the present. The white dot represents the median and the green bar represents the interquartile range.

Predicted distributions differed between present and past conditions (Fig. 2) both in geographical location and probability of occurrence of *P. oceanica*. Predicted habitats under present conditions are located mainly in the Northern Mediterranean Sea and mostly in the Western Mediterranean basin. In contrast, *P. oceanica* could have had a wider distribution in the Southern Mediterranean during the LGM, in separated refugial regions located in the Western, Central and Eastern Mediterranean Sea. Hindcasts obtained with each OGCM differed: CCSM found very low probabilities in the Mediterranean Sea in comparison with CNRM (Supplementary Fig. S1). As a result, the LGM ensemble showed lower overall probability of occurrence than that predicted for current conditions (Figs 2 and 3). Clamping masks revealed some uncertainty in hindcasts located in the north of the study area as the CCSM LGM minimum SSTs are under the values used for training (Supplementary Fig. S2). This uncertainty could affect our hindcast results only in the Mediterranean coasts of France and in the North of the Adriatic Sea, the coldest regions in the Mediterranean Sea during the LGM.

### Comparison of range predictions with genetic diversity and structure of *P. oceanica* populations

**Figure 4:**
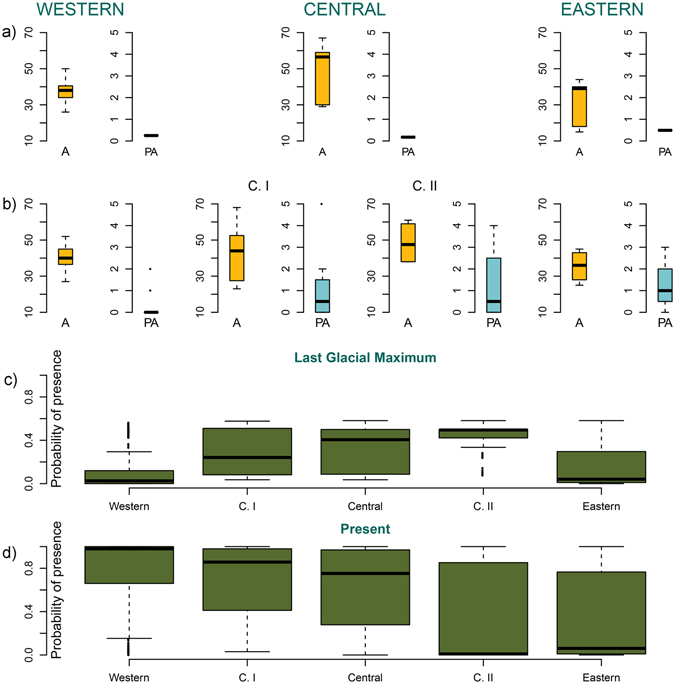
Comparison among the genetic diversity found by (**a**) ref. 15 and (**b**) ref. 17 in the different regions of the Mediterranean Sea, and the probability of occurrence of *Posidonia oceanica* found by this study during the Last Glacial Maximum (LGM) (**c**) and present conditions (**d**). Central regions seem to have been relevant glacial refugia according to the high values found both for allelic richness (A) and the probability of occurrence during the LGM. PA: private alleles; C. I. and C. II.: Central I and Central II subregions of the Central Mediterranean found by ref. 17.

When examining predictions by distinct genetic regions, the Central (both C. I and C. II regions) showed higher probability of presence during the LGM than those for the Western and Eastern Mediterranean (Fig. 4). This is in agreement with the higher allelic richness (A) found by both studies in these Central regions, in comparison with the other regions (mean A = 42.37 in C. I; 49.66 in Central; 48.5 in C. II; see also Fig. 4). Predictions across time showed good conditions for persistence of stable *P. oceanica* populations in the Central Mediterranean Sea.

## Discussion

The cold waters dominating the Mediterranean Sea during the Last Glacial Maximum probably resulted in poorer conditions for the development of *Posidonia oceanica* meadows compared to those found at present. According to our LGM ensemble, SST seems to have been sufficiently warm in the Southern Mediterranean Sea during the LGM to allow persistence of this seagrass species in this region. There, three putative glacial refugia covered separate coastal areas in each of the Western, Central and Eastern Mediterranean Sea, in agreement with the three main genetically structured regions revealed by previous studies^15,17^. Model results suggest that the Central Mediterranean Sea may have constituted the most relevant region allowing the persistence for *Posidonia oceanica* meadows during the Last Glacial Maximum (LGM), as it is the region that showed both the highest probability of occurrence under LGM conditions and where suitable conditions have remained available through present times. The concurrence between our predictions and the higher genetic diversity (allelic richness) found in this Central region further confirms the relevance of the Siculo-Tunisian Strait as a secondary contact zone between western and eastern *P. oceanica* populations, as suggested by ref. 15.

**Figure 5:**
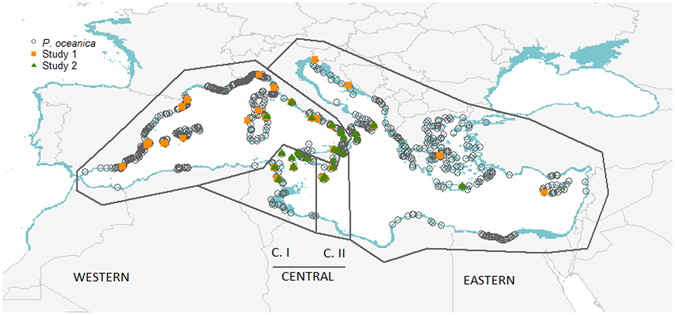
Occurrence data used for ecological niche models of *Posidonia oceanica* (grey circles), and genetic regions identified in the Mediterranean Sea by ref. 15 (Study 1): Western, Central and Eastern (populations in orange squares); and ref. 17 (Study 2): Western, Central I (C. I), Central II (C. II), and Eastern (populations in green triangles). The map was generated using R^43^.

The progressive warming of the Mediterranean waters from the LGM to the present time caused a northwards shift in the distribution of *P. oceanica* (Fig. 2), which nowadays is more abundant in the Northern Mediterranean shores (Fig. 5). The minimum SST was more relevant for delimiting the distribution of *P. oceanica* in our models than the maximum. Although there are differences between the latitudinal limits described by the two Ocean General Circulation Models used, the Northern Adriatic and Aegean sub-basins probably were too cold for the presence of *P. oceanica* during the LGM according to the lowest SST threshold for current occurrence (6.98 °C). Thus, after the LGM, the Northern Mediterranean basin could have experienced a recolonization process by seagrass originating in the southern LGM refugia, from which most of the current meadows derive. In contrast, the maximum SST could not have been a factor impeding the presence of *P. oceanica* anywhere in the Mediterranean during the LGM, as all the area was under the thermal limit (29.21 °C) inferred from current presence patterns.

According to our models, in the past, SSTs were favourable for the species in the majority of the Southern Mediterranean Sea, especially in the Central region (Figs 2 and 4). The Central region showed the highest probability of occurrence during the LGM (Fig. 4) and also showed high probabilities under current conditions. These results are in agreement with previous genetic studies^15,17^, which found in this Central region the highest allelic richness (Fig. 4) over the entire Mediterranean. However, these studies hypothesized the location of probable refugial areas in the eastern and western basins, with a subsequent contact zone in the center. Our niche modelling results now raise a novel hypothesis of long-term persistence in the central zone, which is also in agreement with the existence of unique private genetic diversity in this area that is not merely the result of admixture between the east and the west. Under the assumption that local persistence of the meadows across time could maintain a higher effective population size and harbour more genetic diversity, our results support the hypothesis that the Central region of the Mediterranean basin was likely the most relevant glacial refugium for the preservation of the populations and genetic diversity of *P. oceanica*.

Although our findings support that during the LGM the Central Mediterranean region could have acted as an important refugial area, the main connection between the western and eastern basins (the Siculo-Tunisian Strait) is also an important boundary in the genetic structure of several Mediterranean species besides *P. oceanica* (e.g. refs 5–8). In addition, the Siculo-Tunisian Strait was narrower when sea level was low during the Pleistocene glaciations. At that time as in the present^16^, eventual gene flow between the main Mediterranean basins took place through this Central region, a secondary contact zone between western and eastern populations^15^. As *P. oceanica* is a foundational seagrass, we hypothesize that the existence of this refugial area could have also contributed to the maintenance of associated biota during glacial periods (namely species dependent on *P. oceanica* for habitat and food, such as *Pinna nobilis* and *Sarpa salpa*). However, together with the poorer conditions revealed by our study, there is evidence of meltwater discharges affecting this area after the LGM (from 9.2 ka to 6.5 ka) which affected cold-sensitive planktonic assemblages^26^. Thus, past meadows of *P. oceanica* could have been more scattered than nowadays and might have suffered eventual range contractions until its postglacial colonization was favoured by widespread warming of the Mediterranean basin throughout the Holocene.

The existence of a higher number of private alleles in the Eastern Mediterranean (Fig. 4) suggests long-term persistence there with sufficient time for evolution of unique diversity, in strong contrast with the Western Mediterranean where few private alleles were found to date in the sampled regions. Two alternative hypotheses might explain these data, either bottleneck effects linked with recent colonization time, or the poor sampling in northern Africa to date might have missed older populations (as suggested by our results) that might hypothetically harbour higher diversity in the western basin. However, the scarcity of data on occurrences of this seagrass in the eastern basin, especially the southeastern, suggests that maybe current eastern *P. oceanica* populations are remnants of past more widespread meadows. A northwestern to southeastern decreasing gradient of species richness and nutrient concentrations has been described in the Mediterranean Sea^1,2^. However, although the distributional range of *P. oceanica* appears to reflect a general trend of reduction towards the southeastern basin, similar to other Mediterranean species, unique distinct genetic diversity remains there, in apparently genetically isolated ancient populations, as also found for the seagrass *Cymodocea nodosa*^5^.

Our prediction for the present is quite accurate in terms of sensitivity (correctly predicted presences) and according to the known current distribution of the seagrass^14,27^. The minimum thermal limit found for the species occurrences renders the entire study area suitable in the winter (except the colder Black Sea). However, there is a scarcity of occurrences below 11 °C (see Fig. 1). The species is historically known to be absent in the colder parts of the Mediterranean (e.g. the Gulf of Lion and the westernmost part of the Alboran Sea; see ref. 28). Occurrences which obtained the lowest probability correspond to areas with scattered and fragmented meadows which are occupying marginally high SST conditions (eg. the Eastern Aegean Sea). Some historical records of meadows from Syria and Lebanon in warm waters obtained a low probability and are actually absent nowadays^27,28^. However, these findings may be somewhat limited by the uncertainty on the current distribution of the species, as there is still 46.6% of coastline length without data, most of them (43%) in the eastern basin^27^. Our model, however, also predicts that excessive present summer temperatures already limits the distribution of *P. oceanica* at present in some areas of the Eastern Mediterranean, particularly in the SE region (Fig. 2), consistently with the absence of the species from the shores of Eastern Egypt, Syria and Lebanon^27^. Based on the distribution of *P. oceanica* in the Eastern Mediterranean, a maximum temperature of 28.4 °C was set as the Maximum Tolerable Temperature limiting its distribution^29^. This is consistent with the temperature threshold of 28 °C reported to trigger mortality of *Posidonia oceanica* when exceeded^30^. These observations support the inference here, based on the distribution of maximum seawater temperature in relation to presence/absence of *P. oceanica* across the Mediterranean, of a maximum thermal tolerance of 29.21 °C for this species.

The study is affected by uncertainties associated to the resolution of the input data used, characteristic of efforts to model coastal species. Satellite-derived data for large-scale oceanic variables are not usually available at a resolution higher than 9.2 km (about 0.083° × 0.083°) which could affect the capacity to resolve marine microhabitats. Besides, the availability of LGM oceanic variables is more limited than for current times, thus the set of predictors accessible for hindcasting is reduced in comparison to that available for terrestrial studies. In this study, the use of just two SST variables might also have produced some overprediction. This could have affected mainly the prediction for the North African coast where uncertainty is higher due to the lack of records for most of the countries in the region, a bias also noticed for *Cymodocea nodosa*^31^. Another source of uncertainty could be attributed to the differences that exist between the LGM OGCMs available to produce the hindcasts, a challenge which we have tried to reduce by using an ensemble approach.

*P. oceanica* is experiencing an important decline in meadows in the last 50 years due to local (physical impacts, eutrophication) and global impacts (climate change, invasions)^14,27^. This tendency towards regression affects the areal extent, shoot density and cover of the seagrass^14^, and possibly is already producing a loss in the genetic diversity shown here. The results of this research support the idea that past climate influenced the genetic structure and diversity of *P. oceanica*. But the western basin, which shows the higher probability of occurrence nowadays, is also showing a higher detected decline due to anthropogenic pressures combined with current warming^14^, which is forecasted to become more acute in the future^32^. The fragmentation and disappearance of meadows could produce irreversible genetic bottlenecks which could threaten the survival of key populations and of much of the species gene pool in case of a probable future climate warming, as the poleward migration of the biogeographical range generally displayed by marine species to cope with ocean warming^33^ is precluded, for the case of the endemic Mediterranean species, by the presence of the European continental mass^34,35^. *P. oceanica* has already been considered vulnerable to climate change and a possible extinction of the western *P. oceanica* meadows by 2050 due to increasing frequency and severity of heat waves with climate change has been suggested^32^. More research on forecasting the distribution of this long-lived seagrass could provide insight on the impact of climate warming on the future extent of the species, and help apportion the effects of ocean warming from those of local impacts.

This study has shown that past LGM distribution of *P. oceanica* might have been restricted to the southern Mediterranean Sea as a consequence of the colder LGM northern Mediterranean Sea water. In the South, three glacial refugia have been detected in congruence with previously studied genetic regions. Among them, the most important might be the refugium located in the Siculo-Tunisian Strait, that was previously defined as a transition zone between western and eastern basins, and where populations of *P. oceanica* have been able to preserve their highest genetic diversity.

## Methods

### Species and environmental data

Data on the presence of *P. oceanica* were compiled from an extensive literature review, and were complemented with locations digitized from the seagrass atlas of Spain^36^, and ref. 27. The dataset compiled is available as Supplementary Dataset 1. As just one occurrence for each grid cell was used in the modelling process, the total 1139 records compiled collapsed into 709 presence cells after georeferencing species presence data to a 9.2 km (~0.083° × 0.083°) grid resolution. We considered the entire distributional range of the species (the Mediterranean Sea) and adjacent areas (North-East Atlantic coasts and the Black Sea), using the 30 arc-seconds General Bathymetric Chart of the Oceans (GEBCO; http://www.gebco.net/) to limit the study area to the photic zone (~40 m depth).

We used environmental variables available to represent relevant present and LGM conditions: the minimum, mean and maximum SST in summer and winter, and the average salinity. Variables for current conditions were derived from OSTIA system^37^, while paleoclimate data were obtained from two LGM experiments from the Coupled Model Intercomparison Project (CMIP5): CNRM-CM5 (over 200 years) and CCSM4 (over 100 years). We inferred the LGM’s coastline by calculating a mean sea-level change of −116 m relative to present from bathymetric GEBCO data, consistently with the ice-sheet reconstruction provided by the Paleoclimate Modelling Intercomparison Project Phase III (PMIP 3; http://pmip3.lsce.ipsl.fr/).

### Ecological niche modelling

We first tested correlation among the seven initial variables discarding those showing a Pearson’s product-moment correlation (r) ≥ 0.7 (p < 0.001), to avoid the use of strongly-correlated variables. We implemented an ensemble approach using “biomod2” package^38^ in R. Six presence-absence algorithms were used: generalized additive model (GAM), flexible discriminant analysis (FDA), generalized boosting model (GBM), multiple adaptive regression splines (MARS), generalized linear model (GLM), and random forest (RF). Although “presence” records are unambiguous, the reliability of “absence” data are more difficult to ascertain. If no reliable absence data are available, the method used to obtain “pseudo-absences” strongly conditions the prediction. Here we use a random selection of pseudo-absences to obtain a constrained distribution^39^. Due to the relatively small study area (9,262 grid cells), the same number of pseudo-absences as presence cells were extracted at random, but creating 3 pseudo-absence sets. We split data into a validation (30%) and a calibration set (70%) and performed 10 iterations for each set of pseudo-absences and algorithm. The 180 models obtained (6 modelling techniques x 3 pseudo-absence sets x 10 iterations) were evaluated by means of the area under the receiver operating characteristic (ROC) curve (AUC), ROC-derived specificity (absences correctly predicted) and sensitivity (presences correctly predicted) measures^40^, and the true skill statistic (TSS^41^). We used the threshold at which the sum of the sensitivity and specificity is highest^38^. A “committee averaging” ensemble for present conditions was computed by averaging binary predictions, we used this ensemble approach for its adequate performance on the prediction of other seagrass species^31^. Just those models achieving TSS ≥ 0.55 or AUC ≥ 0.8 were used to produce ensembles. Both ensembles (one per validation measure criteria) were projected to the LGM to produce separate hindcasts derived from the two LGM reconstructions (CCSM and CNRM). As ocean general circulation models (OGCMs) show variability among them, we produced an averaged ensemble computed from both OGCMs hindcasts and validation criteria. Moreover, we computed a clamping mask to identify uncertain locations in our hindcasts using LGM variables which minimum and maximum values were outside the training range.

We assessed the importance of each variable in the ensemble for present conditions by calculating iteratively the correlation between the complete model and the model without one variable (see ref. 42). After performing three permutations of this procedure, variables were ranged from 0 to 1 (the highest importance).

### Genetic diversity data

We compiled data of genetic diversity and structure in *P. oceanica* populations across the Mediterranean Sea from two previous studies^15,17^ and calculated the mean values of allelic richness and private alleles in each genetic cluster identified by them. Reference 15 analyzed 34 populations and identified three regions in the Mediterranean: Eastern, Central and Western; while ref. 17 analyzed genetic diversity of 27 populations and found a total of four, as a finer genetic structure analysis in the Central region revealed that this region was subdivided in two (C. I and C. II; Fig. 5). We extracted the probability of occurrence obtained by the ensembles of averaged binary predictions for current and LGM conditions in relation to the genetic regions identified by these studies.

## Electronic supplementary material

Supplementary Information

Dataset 1
