# Author Correction: Palaeoclimatic conditions in the Mediterranean explain genetic diversity of *Posidonia oceanica* seagrass meadows

**DOI:** 10.1038/s41598-020-62684-7

**Published:** 2020-04-03

**Authors:** Rosa M. Chefaoui, Carlos M. Duarte, Ester A. Serrão

**Affiliations:** 1 CCMAR - Centro de Ciências do Mar, CIMAR Laboratório Associado, Universidade do Algarve, Campus de Gambelas, 8005-139 Faro, Portugal; 2 King Abdullah University of Science and Technology (KAUST), Red Sea Research Center (RSRC), Thuwal, 23955-6900 Saudi Arabia

Correction to: *Scientific Reports* 10.1038/s41598-017-03006-2, published online 02 June 2017

A supplementary file containing Dataset 1 was omitted from the original version of this Article. This has been corrected in the HTML version of the Article; the PDF version was correct at time of publication.

