# Supplementary material for: Palaeoclimatic conditions in the Mediterranean explain genetic diversity of Posidonia oceanica seagrass meadows
Source: Sci Rep. 2017 Jun 2;7:2732. doi: 10.1038/s41598-017-03006-2 (PMC5457430; doi:10.1038/s41598-017-03006-2)
Supplement: Supplementary file 1 — Supplementary Information [file 41598_2017_3006_MOESM1_ESM.pdf]

SUPPLEMENTARY INFORMATION

Palaeoclimatic conditions in the Mediterranean explain genetic diversity of *Posidonia oceanica* seagrass meadows

Rosa M. Chefaoui[1*], Carlos M. Duarte[2], Ester A. Serrão[1]

[1.] CCMAR - Centro de Ciências do Mar, CIMAR Laboratório Associado, Universidade do Algarve, Campus de Gambelas, 8005-139 Faro, Portugal

[2.] King Abdullah University of Science and Technology (KAUST), Red Sea Research Center (RSRC), Thuwal, 23955-6900, Saudi Arabia

Table S1. Mean validation scores of models obtained for *Posidonia oceanica*. Just those with AUC ≥ 0.9 or TSS ≥ 0.65 were used to produce the ensemble. AUC: the area under the receiver operating characteristic (ROC) curve; TSS: the true skill statistic; GLM: generalized linear model; GAM: generalized additive model; FDA: flexible discriminant analysis; GBM: generalized boosting model; MARS: multiple adaptive regression splines; RF: randomForest.

**Model**	**AUC**	**TSS**	**Sensitivity**	**Specificity**
GLM	0.80 ± 0.01	0.50 ± 0.02	87.40 ± 3.36	64.11 ± 5.27
GBM	0.83 ± 0.01	0.55 ± 0.01	86.23 ± 3.61	69.78 ± 4.60
GAM	0.80 ± 0.01	0.50 ± 0.02	87.94 ± 6.80	63.36 ± 7.87
FDA	0.81 ± 0.01	0.54 ± 0.02	87.57 ± 2.64	66.02 ± 4.37
MARS	0.82 ± 0.01	0.54 ± 0.02	88.90 ± 3.82	64.80 ± 5.84
RF	0.82 ± 0.01	0.53 ± 0.02	85.78 ± 3.86	69.05 ± 5.13

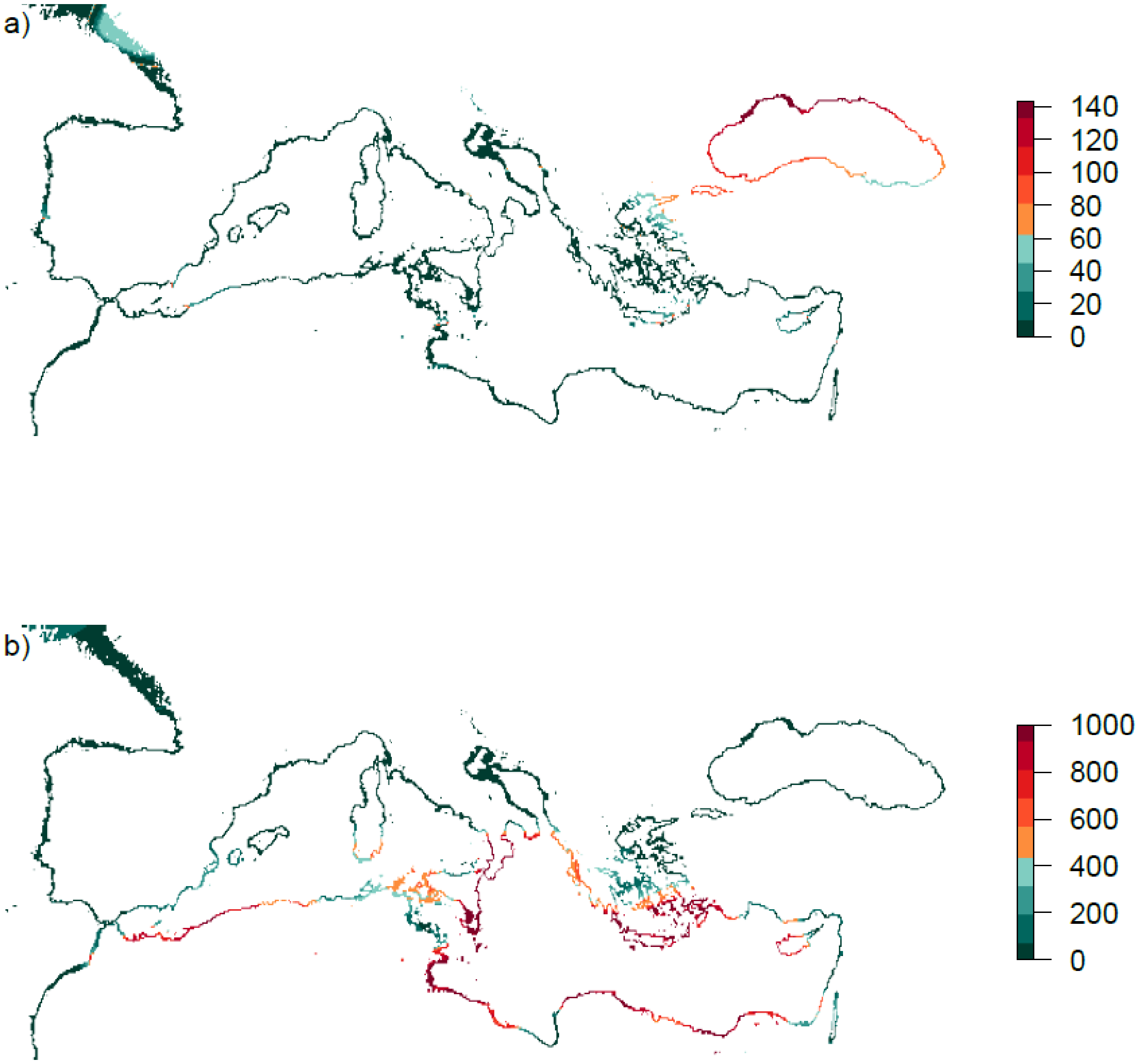

Fig. S1 Hindcasts obtained from the two Ocean General Circulation Models (a) CCSM; b) CNRM) used afterwards for the Last Glacial Maximum ensemble for *Posidonia oceanica*. Maps were generated using R [43].

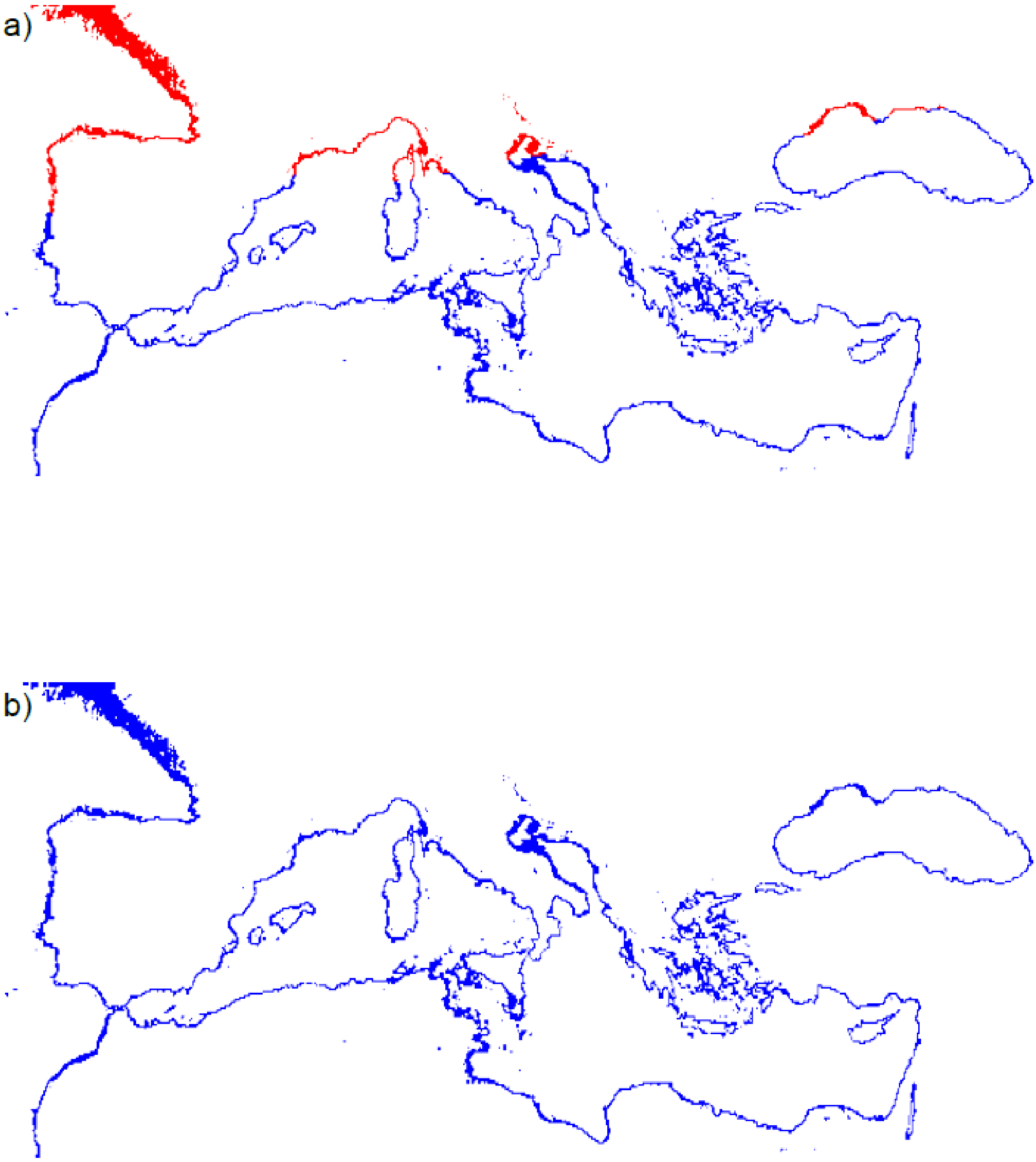

Fig. S2 Clamping masks for the two ocean general circulation models: CCSM (a) and CNRM (b). Uncertainty in predictions due to variables outside the range used for training the models are shown in red. Maps were generated using R [43].
